# Enhancing Ophthalmic Care: The Transformative Potential of Digital Twins in Healthcare

**DOI:** 10.7759/cureus.76209

**Published:** 2024-12-22

**Authors:** Raphael G Banoub, Harshal Sanghvi, Gurnoor S Gill, Alfredo A Paredes, Harnaina K Bains, Anita Patel, Ankur Agarwal, Shailesh Gupta

**Affiliations:** 1 Department of Ophthalmology, Broward Health, Fort Lauderdale, USA; 2 Department of Technology and Clinical Trials, Advanced Research, Deerfield Beach, USA; 3 Department of Medicine, Florida Atlantic University Charles E. Schmidt College of Medicine, Boca Raton, USA; 4 Department of Clinical Trials, Advanced Research, Deerfield Beach, USA; 5 Department of Ophthalmology, Florida Atlantic University Charles E. Schmidt College of Medicine, Boca Raton, USA; 6 College of Electrical Engineering and Computer Science (CEECS), Florida Atlantic University, Boca Raton, USA

**Keywords:** digital health technology, digital twin, ophthalmology, surgery, virtual reality

## Abstract

This literature review explores the emerging role of digital twin (DT) technology in ophthalmology, emphasizing its potential to revolutionize personalized medicine. DTs integrate diverse data sources, including genetic, environmental, and real-time patient data, to create dynamic, predictive models that enhance risk assessment, surgical planning, and postoperative care. The review highlights vital case studies demonstrating the application of DTs in improving the early detection and management of diseases such as glaucoma and age-related macular degeneration. While implementing DTs presents challenges, including data integration and privacy concerns, the potential benefits, such as improved patient outcomes and cost savings, position DTs as a valuable tool in the future of ophthalmic care. The review underscores the need for further research to address these challenges and fully realize the potential of DTs in clinical practice.

## Introduction and background

Virtual healthcare has rapidly evolved, offering a transformative approach to delivering medical services through remote consultations and digital tools [1]. Initially aimed at bridging geographical gaps in healthcare access, the adoption of virtual healthcare has been significantly accelerated by the COVID-19 pandemic, highlighting its critical role in maintaining healthcare continuity amidst physical distancing measures [2]. Virtual healthcare encompasses a variety of services, including telemedicine, remote patient monitoring, and mobile health applications, all designed to facilitate patient care without the absolute need for physical presence [2].

The widespread adoption of virtual healthcare has brought several notable benefits. It has enhanced access to medical services, particularly for remote or underserved patients, reducing healthcare delivery disparities [2]. Moreover, virtual healthcare has proven cost-effective, reducing the need for physical infrastructure and enabling more efficient use of medical resources [1]. Patients benefit from the convenience of receiving care from the comfort of their homes, which has been particularly valuable during the COVID-19 pandemic, reducing the risk of virus transmission [1]. However, the rapid implementation of virtual healthcare has also highlighted several challenges. The digital divide remains a significant barrier, with disparities in access to technology and internet connectivity affecting the equitable distribution of virtual healthcare services [2]. Additionally, data privacy and security concerns have emerged as the increased use of digital platforms necessitates robust measures to protect patient information [2]. Privacy laws such as the Personal Information Protection and Electronic Documents Act (PIPEDA) and health information statutes must be adhered to, ensuring that virtual care complies with stringent privacy regulations [2].

In the field of ophthalmology, digital healthcare has demonstrated significant potential. Automated deep-learning algorithms have been developed to analyze ocular images, enabling more accurate and efficient diagnoses [3]. These advancements have facilitated the early detection of eye diseases such as diabetic retinopathy and age-related macular degeneration, potentially improving patient outcomes [3]. Digital twins (DTs) in ophthalmology, virtual replicas of a patient's ocular structures, allow for personalized treatment plans and predictive disease progression modeling [4]. Despite these promising developments, the integration of digital healthcare in ophthalmology also faces challenges. Ensuring the accuracy and reliability of digital tools is paramount, and there is a need for ongoing research to validate these technologies and address any biases [4]. Additionally, the acceptance and adoption of digital healthcare by patients and healthcare providers will be crucial for its sustained success in ophthalmology [3].

DT is a dynamic, virtual representation of a physical object, system, or environment that integrates real-time data, simulations, and analytics to reflect its current accurately and predicted states [5]. It constantly updates through data from sensors, internet of things (IoT) devices, and other sources, enabling predictive modeling, performance optimization, and lifecycle management. By merging detailed geometry, physical properties, and operational conditions, DTs allow organizations to test scenarios, anticipate failures, reduce downtime, and improve efficiency. They are leveraged across industries, from manufacturing and energy to healthcare and smart cities, to enhance decision-making, streamline maintenance, and foster innovation in a safe, virtual environment. This allows for the simulation of dynamic entities through a virtual model [5]. Therefore, DT can be a predictive tool for disease diagnosis and progression, especially when combined with machine learning, for greater decision-making capabilities [5]. The combination of DT with artificial intelligence (AI) algorithms is known as an “intelligent twin,” which can perform self-adaptation via real-time updates and utilize AI for learning and reasoning skills [5]. By constantly communicating with the real world, intelligent DTs can predict the future state of the real system and thus facilitate diagnosis and allow for pre-emptive clinical decisions [5].

DTs can be used to model structures ranging from the human cell to the entire human body [5]. The concept of an intelligent twin has promising applications in healthcare by simulating patient-specific models, which can help predict disease progression and treatment outcomes, offering personalized healthcare solutions [6]. For example, intelligent DTs are being used in healthcare to emulate specific organs and model their disease states, such as heart models for pacemaker performance prediction. Similar DT organ models were implemented for the lungs during the COVID-19 pandemic to improve outcomes [5]. These organ models also allow for faster treatment and device design as innovators can use DTs to rapidly test products [5]. In the field of surgery, DT is being used to help surgeons practice techniques and decide optimal postoperative treatment based on a patient’s individual characteristics and surgery [5].

The purpose of this study is to explore the transformative potential of DT technology in ophthalmology and its capacity to advance personalized medicine. By integrating genetic, environmental, and real-time patient data, DTs create predictive, dynamic models that can assist clinicians in risk assessment, treatment planning, and disease management. The study aims to demonstrate how these virtual replicas enhance early detection of ophthalmic diseases like glaucoma and age-related macular degeneration, optimize surgical strategies, and refine postoperative care. The hypothesis driving this research is that incorporating DT technology in ophthalmology can lead to improved patient outcomes, enhanced precision in medical interventions, and cost-effective healthcare delivery while addressing the challenges of data integration and privacy.

We conducted a review search using PubMed and Web of Science to identify peer-reviewed literature on several DT applications in ophthalmic healthcare. Studies were included if they integrated genetic, demographic, or physiological patient data into DT models and addressed predictive modeling, disease management, surgical planning, or personalized medicine in ophthalmology. Priority was given to research presenting case studies or patient-specific results that demonstrated clinical impact. We excluded publications that did not focus on the ophthalmic domain, were unrelated to healthcare, or addressed purely technical aspects of DTs without clinical application. Furthermore, the search strategy focused on identifying studies published between 2015 and 2024 from databases such as PubMed, Scopus, and Web of Science, using keywords including "Digital Twin", "Ophthalmology", "Personalized Medicine", and "Artificial Intelligence". Inclusion criteria prioritized peer-reviewed articles on DT applications in healthcare, specifically ophthalmology, with case studies, clinical trials, and narrative reviews that integrated patient-specific data, such as genetic profiles and imaging. Studies published before 2015, lacking relevance to DT technology, or focused on non-healthcare fields were excluded. This approach ensured a focus on recent, high-quality literature with actionable insights into DT applications in ophthalmology.

## Review

Assessing risk and disease before diagnosis

DTs utilize a comprehensive array of patient data, including electronic health records (EHRs), wearable devices, and real-time physiological monitoring, to accurately represent an individual's health. By continuously integrating and updating these data sources, DTs offer a real-time reflection of a patient’s health status, allowing for precise monitoring and early detection of disease markers [7]. This dynamic integration enables healthcare providers to identify subtle changes in a patient's health that may indicate the onset of a disease, facilitating early interventions that can significantly alter the course of treatment and outcomes [8].

The inclusion of genetic information into DTs significantly enhances their predictive capabilities by enabling the identification of hereditary risks and susceptibilities. For example, when combined with an individual’s medical history and lifestyle information, genetic data creates a comprehensive risk profile that can be used to tailor preventive measures and treatment plans [9]. This is particularly evident in functional medicine, where DTs can simulate patient-specific responses to various treatment options [10].

Functional medicine, an individualized and science-based approach, emphasizes addressing the root causes of diseases by considering genetic, environmental, and lifestyle factors. This integrative approach is gaining prominence in ophthalmology as systemic health and cellular functions are increasingly linked to eye diseases. DT technology, which creates virtual replicas of patients, can enhance these functional approaches by simulating disease progression and treatment outcomes. For example, in myopia progression, DTs could model calcium signaling in retinal horizontal cells, enabling personalized interventions to prevent the worsening of the condition [5]. Similarly, DTs could simulate gut microbiota dynamics and their systemic effects, offering tailored dietary or probiotic treatments to reduce inflammation and mitigate diabetic retinopathy risk [10]. Nutritional strategies for retinal health, including lutein, zeaxanthin, and omega-3 supplementation, could be optimized using DTs to predict how specific nutritional changes impact retinal function and disease progression in individuals [11]. In managing chronic dry eye, DTs could model tear film dynamics and inflammatory responses to identify the most effective anti-inflammatory diets or supplements. Systemic health management, such as optimizing glycemic control in diabetes, could be enhanced by DTs simulating the effects of interventions on ocular outcomes like diabetic retinopathy or uveitis. With advancements in AI and imaging technologies, DTs further elevate the integration of functional medicine in ophthalmology, enabling precise diagnostics, early identification of systemic conditions, and personalized interventions that address root causes. Functional ophthalmology offers a revolutionary approach to understanding and managing eye conditions by combining DT simulations with systemic health optimization [6].

This integration minimizes the risks associated with treatments by avoiding less suitable therapies, thereby improving overall patient outcomes [12]. Genetic data are integrated into the DT model to identify mutations associated with hereditary pathologies [13]. For example, specific genes, such as CYP1B1, which is linked to primary congenital glaucoma, and MYOC, OPTN, and TBK1, which are associated with primary open-angle glaucoma (POAG), are highlighted as critical markers for assessing genetic risk [14]. By incorporating this genetic information, the DT enhances the accuracy of risk assessments, enabling early detection of these conditions before clinical symptoms emerge [13]. This integration aligns with the study's objective of using DTs to simulate patient health scenarios and predict disease progression based on genetic predispositions [13].

Environmental factors, such as lifestyle choices, socioeconomic status, and exposure to environmental toxins, are critical in the development and progression of diseases. DTs incorporate these factors alongside genetic and physiological data to provide a more accurate prediction of disease likelihood. By simulating how various environmental conditions might interact with a patient's genetic and health profiles, DTs can highlight potential risks that might not be apparent through genetic or clinical data alone [8]. This capability is particularly important for diseases influenced by external factors, such as vernal keratoconjunctivitis being more common in hot and dry areas, where the interplay between environment and health is significant [9].

The predictive power of DTs is greatly enhanced by applying machine learning algorithms. These algorithms analyze vast datasets to uncover patterns and correlations that traditional analysis methods might miss. For instance, by applying machine learning to datasets that combine genetic information, patient history, and environmental factors, DTs can predict the onset of diseases like diabetes or cancer with greater accuracy [7]. Moreover, these systems are designed to continuously learn and update their models as new data becomes available, ensuring that predictions remain accurate and relevant over time [8]. This continuous learning aspect is essential for maintaining the utility of DTs in dynamic healthcare environments where patient data can change rapidly [7]. This adaptability introduces the risk of a "vicious cycle of data contamination," where AI-generated outputs, whether accurate or hallucinated, are later used as training data. This feedback loop can lead to the reinforcement of inaccuracies and the gradual degradation of model reliability. To mitigate this, several strategies are essential. Rigorous data validation by human experts is crucial to ensure that only clinically accurate and relevant information is incorporated into training datasets. Metadata tagging can help distinguish between AI-generated outputs and verified clinical data, allowing models to prioritize reliable information during retraining. To further reduce risks, feedback loops with human oversight are necessary to correct errors and refine the AI’s understanding. Additionally, isolating AI-generated data from primary datasets ensures that models do not self-train on unverified outputs [6]. Advanced bias and hallucination detection systems can flag and filter problematic data before they enter the training pipeline, while periodic audits of the model’s learning patterns and outputs can identify and rectify errors over time. Leveraging explainable AI (XAI) techniques ensures transparency, enabling users to understand how models reach their conclusions and detect potential inaccuracies more effectively [7]. XAI encompasses processes and tools that make AI systems transparent, interpretable, and understandable by revealing the reasoning and factors behind their decisions, thereby improving accountability, trust, and auditability. In healthcare and ophthalmology, XAI ensures clinicians can understand AI-assisted diagnoses or treatment recommendations by highlighting relevant features, such as retinal abnormalities for diabetic retinopathy, and justifying personalized interventions in a clear, trustworthy manner [5]. By integrating these measures, the adaptability of AI systems, such as DTs, can be harnessed to their full potential while avoiding the pitfalls of self-reinforced inaccuracies in healthcare applications.

Recent advancements in DT technology have demonstrated its effectiveness across various medical fields. Cardiovascular applications, like the HeartFlow Analysis and FEops HEARTguide, utilize imaging and computational modeling to support non-invasive diagnostics, optimize surgical planning, and enhance device fitting for conditions like coronary artery disease and structural heart disease. These examples illustrate the precise and diverse uses of DTs in improving medical outcomes across specific conditions [8]. In oncology, DTs predict radiation therapy outcomes by integrating patient-specific data, including MRI or CT imaging, genomic profiles, and tissue microenvironment characteristics, to create personalized simulations of tumor behavior and treatment response. These twins employ advanced computational models such as finite element analysis (FEA) to simulate physical stress and tissue deformation, Monte Carlo methods for precise radiation particle interaction modeling, and machine learning algorithms to predict tumor response based on historical and patient-specific data​ [7]. Additionally, fluid dynamics models analyze vascular effects, and adaptive mesh refinement techniques ensure high-resolution simulations of complex anatomical structures. This combination allows for the optimization of radiation dose distribution, assessment of risks to surrounding healthy tissues, and the creation of tailored treatment plans for individual patients ​[7]. These real-world applications underscore the potential of DTs to transform personalized medicine by integrating and analyzing a wide range of data sources to produce actionable insights [9]. Despite the transformative potential of DTs, several challenges impede their widespread adoption in healthcare. Data integration remains a significant hurdle, as healthcare data are often siloed across different systems and formats, complicating the creation of a unified DT model [7]. Additionally, the use of vast amounts of sensitive patient data raises substantial privacy and security concerns, necessitating robust measures to protect this information and maintain patient trust [8]. Addressing these challenges will require ongoing advancements in data interoperability standards, as well as the development of ethical frameworks to ensure the responsible use of DTs in healthcare [9].

**Figure 1:**
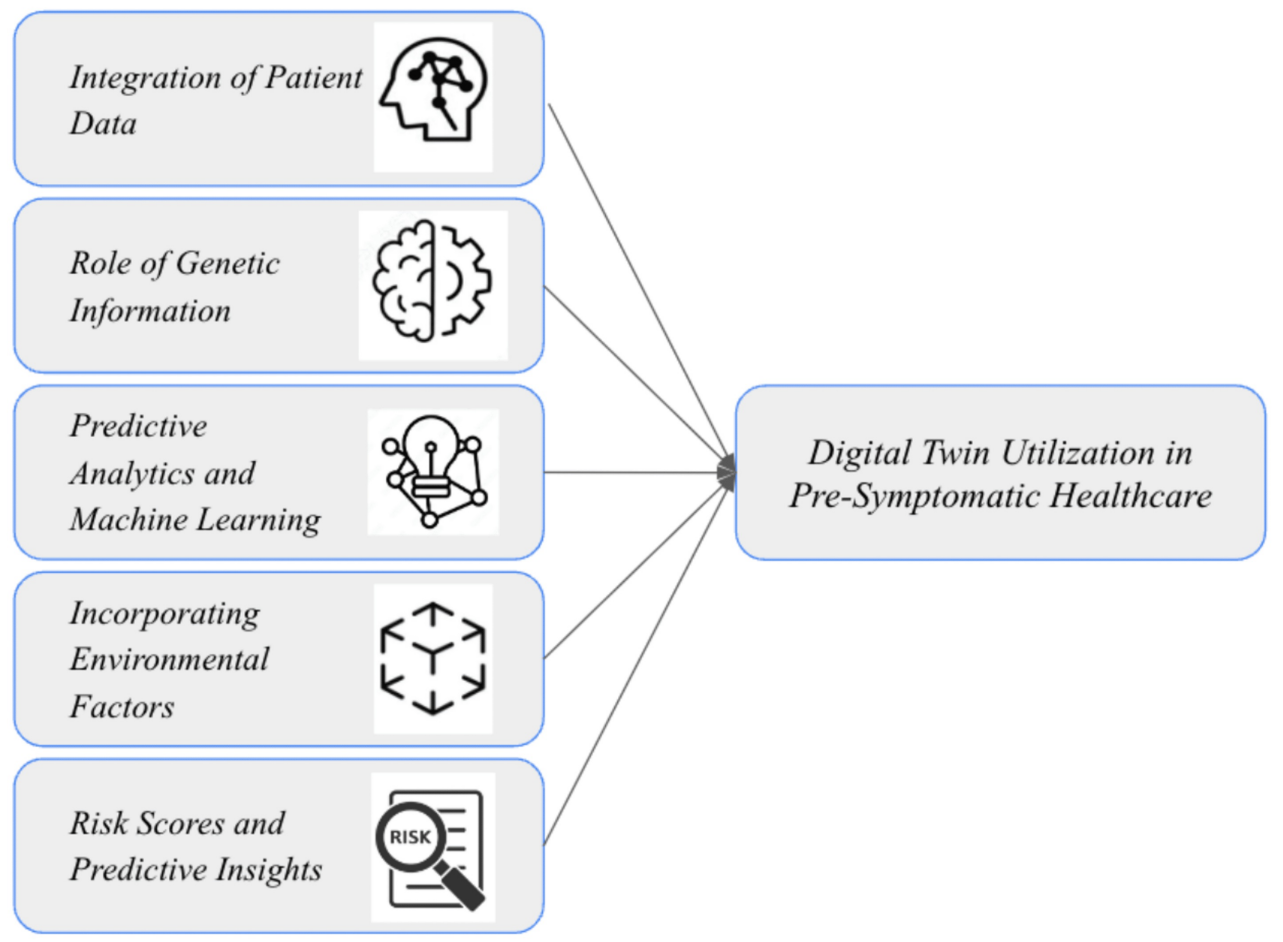
Key components of digital twin utilization in pre-symptomatic healthcare. Figure credit: Gurnoor Gill

In ophthalmology, DT technology has been employed to integrate genetic predispositions, lifestyle factors, and comorbid conditions for comprehensive risk assessments of diseases such as age-related macular degeneration (AMD) and glaucoma [10,11]. For AMD, polygenic risk scores (PRS) based on genetic variants, such as ARMS2 and CFH, are combined with real-time data on lifestyle choices, such as smoking and diet, to predict disease progression. Similarly, for glaucoma, demographic parameters, including age, race, and gender, are integrated into DT frameworks [12,13]. For example, studies highlight that individuals of Black or African American descent, influenced by both genetic and environmental factors like lifestyle choices, face a higher risk of developing glaucoma [14]. These DTs dynamically model these combined influences, offering actionable insights into disease risk and progression while supporting preventive care and personalized treatment strategies. By continuously updating with real-time patient data, the DT framework ensures precision in tailoring interventions and addressing complex interdependencies among genetic, environmental, and clinical factors, as shown in Figure 1.

Managing treatment within the disease

DTs represent a significant advancement in personalized medicine by enabling real-time simulation of patient responses to various treatments. These simulations provide more accurate predictions than traditional statistical methods. DTs utilize a combination of AI, IoT, and closed-loop optimization (CLO) to continuously monitor and adjust treatment protocols. This continuous feedback loop facilitates the refinement of drug dosages and combinations, allowing for personalized treatments tailored to individual patient's unique biological profiles [9]. By integrating data from patient records, biological sources, and mobile sensors, DTs can forecast patient reactions to medication, behavior changes, and environmental factors, which enhances the ability to proactively prevent diseases and optimize treatments [9]. A critical advantage of DTs is their ability to reduce the risk of adverse side effects. The real-time predictive capabilities of DTs allow healthcare providers to fine-tune therapies to the patient's specific needs, minimizing potential side effects. DT technology offers transformative potential in personalizing treatment pathways by integrating patient-specific molecular, phenotypic, and environmental data. These digital models simulate various therapeutic scenarios, allowing healthcare providers to optimize drug dosages and combinations while minimizing the risk of side effects. By leveraging high-resolution computational models, DTs can identify the most effective therapies for individual patients, addressing the complexities of diseases and improving treatment outcomes [15]. Real-time monitoring capabilities enable continuous adjustments to therapeutic plans, ensuring that interventions remain effective and safe over time. This adaptability is particularly valuable in ophthalmology, where DTs can simulate treatment effects for combinations of therapeutic agents, such as eye drops, before application in real-world settings [16].

According to Venkatesh et al., DTs leverage AI, IoT, and CLO to offer predictive capabilities that surpass traditional methods. These systems can model and test different treatment combinations in a virtual environment, allowing clinicians to identify the most effective therapy with the fewest side effects. The use of DTs in ophthalmology could significantly reduce the trial-and-error approach typically associated with determining the optimal combination of eye drops or other therapeutic agents [9].

DTs in healthcare, as detailed by Suchetha et al., enable continuous monitoring and adjustment of treatment protocols [16]. DTs utilize AI, IoT, and CLO to surpass traditional methods of treatment planning and execution. Traditional approaches often relied on retrospective analyses or static predictive models, which lacked adaptability to real-time data changes and individualized patient conditions. In contrast, DTs integrate AI to enhance predictive analytics, IoT for continuous data collection and synchronization, and CLO to refine treatment plans iteratively. For instance, as detailed in Suchetha et al.'s comprehensive review, AI algorithms process heterogeneous data streams to enable precise predictions of treatment outcomes. IoT ensures the seamless flow of physiological and environmental data, creating dynamic patient models. CLO complements this process by iteratively testing and optimizing therapeutic scenarios in virtual environments, ensuring the best clinical outcome with minimized side effects. This convergence not only reduces the trial-and-error nature of traditional therapies but also allows for real-time adjustments based on the patient’s evolving condition. For example, in prostate cancer management, DTs have been used to simulate combinations of hormone therapy, radiation, and surgery to optimize therapeutic efficacy and reduce adverse effects, a functionality that can be extended to ophthalmology to refine regimens for complex conditions like glaucoma or macular degeneration. Björnsson et al. emphasize how DTs construct high-resolution computational models of individual patients, enabling clinicians to virtually test thousands of drug combinations and select the optimal regimen for conditions like glaucoma [15]. In a specific case discussed by Suchetha et al., a DT was utilized for managing prostate cancer, employing machine learning techniques like logistic regression (LR) and recurrent neural networks (RNN) to simulate various treatment pathways, including hormone therapy, radiation, and surgical interventions. This enabled the medical team to evaluate treatment outcomes, optimizing tumor control and minimizing side effects such as incontinence and erectile dysfunction. The machine-learning-based DT system predicted biochemical recurrence (BCR) and assessed long-term risks to enhance decision-making in selecting the most effective treatment strategy [16]. Similarly, in retinoblastoma, DTs could integrate tumor genetics, imaging data, and patient-specific clinical parameters to simulate treatment pathways such as systemic or intra-arterial chemotherapy, radiation therapy, or enucleation. The prostate cancer case underscores how DT technology's adaptability in integrating patient-specific data and treatment simulations can be effectively applied to managing retinoblastoma, ensuring personalized and optimized care [16].

Traditional technologies in ophthalmology, such as static imaging systems (e.g., optical coherence tomography (OCT) and fundus photography) and population-based predictive models, rely on retrospective data analysis and lack the real-time adaptability of DTs. For example, static imaging provides snapshots of a patient's condition but cannot simulate disease progression or test treatment interventions in a dynamic environment. In contrast, DTs integrate real-time data through IoT devices and employ AI to model patient-specific responses, offering a predictive and proactive approach [16]. While traditional models may offer generalized insights, they are limited in their ability to individualize care, particularly for complex diseases like glaucoma or age-related macular degeneration. DTs overcome these limitations by incorporating continuous feedback loops (CLO) to refine therapeutic strategies iteratively, ensuring precision and minimizing adverse outcomes [15]. This real-time adaptability highlights the unique advantage of DTs in ophthalmology and justifies their adoption over existing static approaches [17].

**Figure 2:**
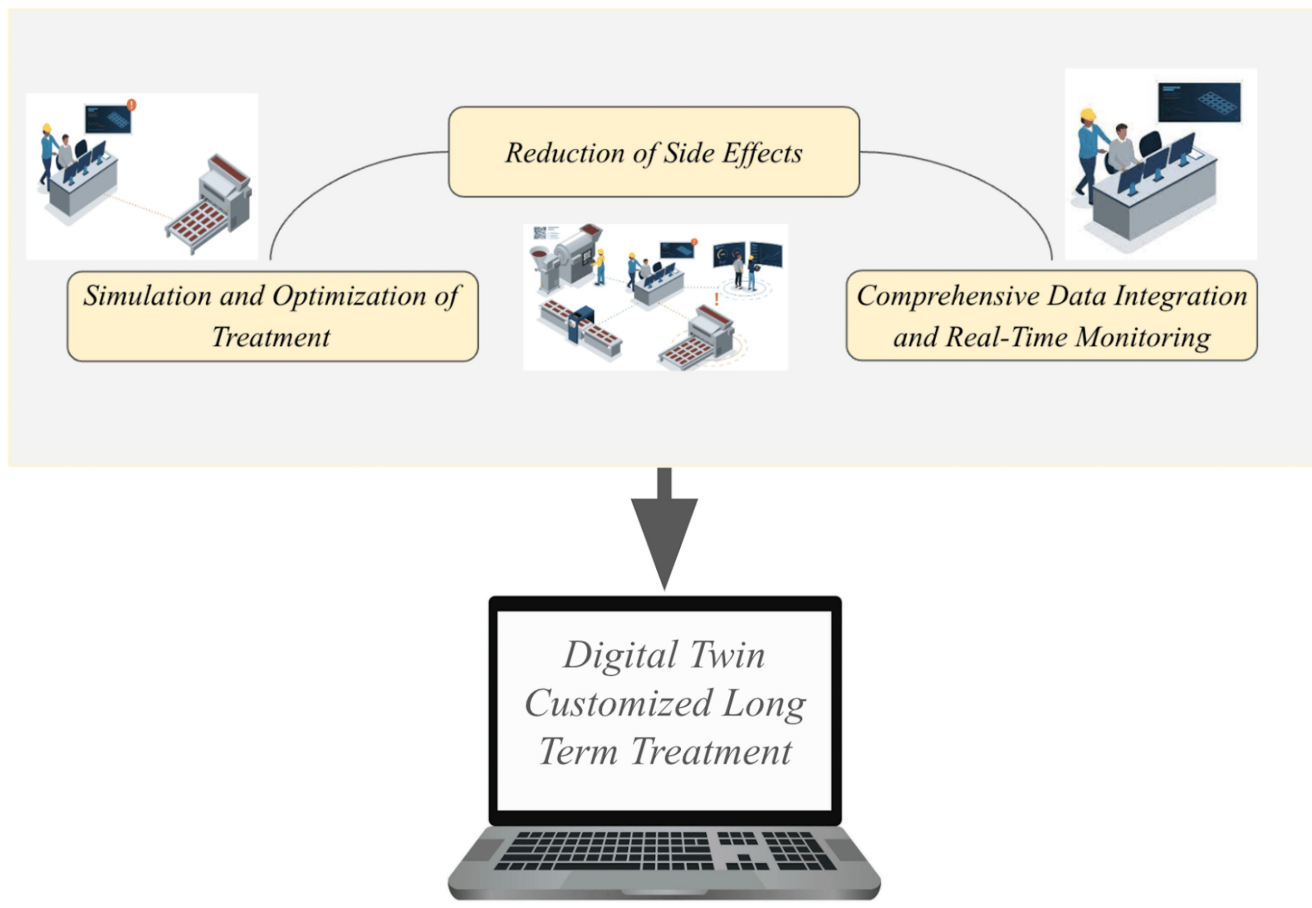
Digital twin-driven customized long-term treatment. Figure credit: Gurnoor Gill

According to Iliută et al., DTs can support early diagnosis and predict the evolution of eye diseases after treatment by providing customized simulation scenarios, as shown in Figure 2. This capability allows clinicians to explore various treatment options in a virtual environment before applying them to the patient, reducing the risk of adverse effects and enhancing treatment efficacy [13].

Digital twin in surgery

Ahmed et al. discuss the emerging role of DTs in surgical practice, highlighting their potential to revolutionize pre-operative planning. DTs allow the creation of patient-specific models that surgeons can use to plan, simulate, and practice procedures in a virtual environment before the actual surgery [17]. This capability helps anticipate complications by enabling surgeons to visualize the patient’s anatomy in detail and refine their surgical approach, thus improving precision [17]. The study also emphasizes that integrating DTs with virtual reality (VR) platforms enhances surgical training and decision-making by offering a realistic environment tailored to each patient’s anatomy [17].​

Croatti et al. discuss the integration of DTs with agent-based systems in healthcare, including a case study on trauma management. Although the focus is on managing acute conditions, the principles of using DTs to simulate and plan procedures are applicable to preoperative contexts [18]. The study explains how DTs can be used to monitor and simulate the patient’s condition in real time, enabling better preparation and planning for surgical interventions. This approach supports surgeons in visualizing the surgical process, anticipating potential complications, and making informed decisions during surgery [18].

**Figure 3:**
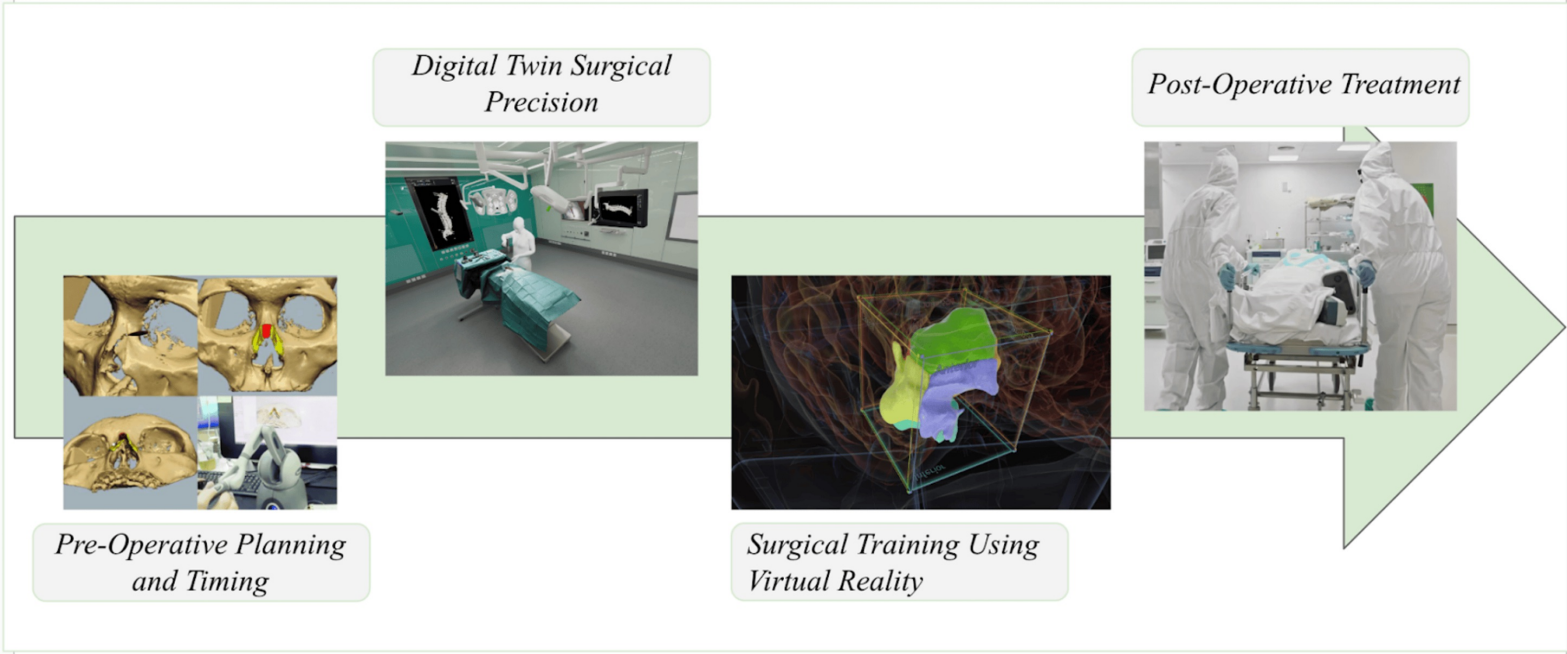
Digital twin in surgical precision and postoperative treatment. Figure credit: Gurnoor Gill

The Twin-S framework is a DT environment specifically designed for skull base surgeries. It enables real-time updates of the virtual model based on the actual surgical progress, helping surgeons visualize the surgical environment and anticipate complications [19]. This allows for precise planning and timing of the surgery, ensuring that the intervention occurs at the optimal moment. The framework combines high-precision optical tracking and real-time simulation to accurately model critical components, such as the patient's anatomy and surgical tools [16]. This system enhances the surgeon's situational awareness and aids in making informed decisions about the timing of the surgery [19]. DTs have been proposed for optimizing surgical timing in arthroscopic knee surgeries. By creating patient-specific virtual models that incorporate detailed 3D anatomy and material properties, surgeons can practice and refine their techniques before the actual surgery [20]. This approach increases the surgeon's confidence and reduces the risk of intraoperative complications [20]. The ability to simulate the procedure in advance provides flexibility in choosing the most opportune time for the surgery, particularly in non-critical cases where timing can significantly impact outcomes. This application is seen as a natural evolution of existing surgical simulation systems, enhancing the ability to plan interventions effectively [20], as shown in Figure 3.

Qin et al. explore the integration of DT technology into computer-assisted surgery (CAS), particularly in the context of preoperative planning. They describe how DTs create high-fidelity virtual models of patients' tissues and organs, which surgeons can use to simulate and plan surgeries with greater accuracy [21]. These models allow for the visualization of dynamic changes during surgery, helping surgeons anticipate complications and improve precision [21]. The study also discusses the application of DTs in remote surgeries, where real-time updates and feedback from the DT guide the surgical process, ensuring better outcomes [21].

In the study by Pajic et al., Optimeyes software was employed to create patient-specific finite element models based on preoperative measurements. The software simulated the effects of strontium-/yttrium-90 beta-irradiation used to treat pterygium, a condition characterized by a growth on the cornea that can lead to astigmatism [22]. The Optimeyes software allowed for accurate predictions of postoperative corneal shape, particularly in terms of astigmatism and corneal curvature changes [22]. This simulation enabled clinicians to understand the biomechanical effects of surgery and to anticipate possible complications like induced corneal astigmatism before performing the procedure [22]. The study highlighted that the software's predictions closely matched the clinical outcomes observed during follow-up, demonstrating its effectiveness in preoperative planning and its potential to improve surgical precision and patient outcomes [22].

A study published in the *International Journal of Medical Robotics and Computer Assisted Surgery* discusses how robotic surgery platforms utilize patient-specific data to create a virtual model of the surgical site [23]. This model acts as a DT, allowing the surgeon to plan and simulate the procedure before making any incisions. The study highlights how this technology reduces the margin of error and improves patient outcomes by enabling more accurate surgical interventions [23].

VR refers to a completely computer-simulated environment that completely immerses the user [24]. VR systems allow users to perform actions in a virtual world, and thus often require hardware devices such as headsets, controllers, or movement trackers [24]. On the other hand, augmented reality (AR) refers to artificial elements being added to a real-world environment to modify reality [24]. AR allows for real-time interaction between the real environment and a simulated environment thus enhancing the user’s experience of the real world [24]. Additionally, while VR typically allows for interaction with completely virtual objects, AR allows for interactions with real, often virtually modified, objects in the environment [24]. VR and AR have broad applications in the field of medicine, especially in anatomical structure education, surgical training, and planning [24].

Research published in *The Lancet Digital Health* explores the use of AR in surgery as a DT by enabling real-time interaction with patient-specific anatomical models during procedures [25]. The study demonstrated that AR systems could overlay 3D models onto the patient’s body, allowing surgeons to see beneath the skin and plan their actions with greater accuracy [25]. The authors concluded that AR could significantly reduce surgical errors and improve patient outcomes by providing a more comprehensive view of the operative field [25].

A study by Zhang et al. assessed the effectiveness of a grape-based microsurgical training course for ophthalmology residents and compared it with VR training modules. The study was designed as a randomized controlled trial including 83 first-year ophthalmology residents with no prior microsurgical experience [26]. Participants were randomized into two groups: group A, which received a four-hour training program using grapes as a model for microsurgery, and group B, which used VR simulators and silicone suture pads [26]. Performance in corneal suturing and circular capsulorhexis on porcine eyes was evaluated post training [26].

The study concluded that grape-based training models are as effective as VR simulators and silicone suture pads for teaching basic microsurgical skills to ophthalmology residents. Furthermore, grape-based models offer advantages such as lower economic cost and easier availability [26]. The economic analysis revealed that the grape-based training model had a significantly lower cost compared to the VR simulator-based training [26].

Furthermore, a study by Leitritz et al. critically evaluated the usability of augmented reality ophthalmoscopy (ARO) for training medical students in BIO. The study involved 37 medical students in their fourth year of medical school, who were randomly assigned to either conventional ophthalmoscopy (CO) training or ARO training using the Eyesi indirect system [27]. Each student received a short introduction to the theoretical principles of indirect ophthalmoscopy and a demonstration by an experienced instructor. Students in the CO group practiced on fellow students with dilated pupils, while those in the ARO group used the Eyesi indirect simulator, which included a headset with a built-in binocular video display and a dummy patient face [27]. After training, all students examined the same real person and drew the optic disk. The performance of the students was evaluated subjectively by three experienced ophthalmologists and objectively using an angular spacing overlay method [27]. The study found no significant differences in the questionnaire responses between the CO and ARO groups regarding efficiency, self-assurance, time constraints, mental stress, and self-evaluation of skills [27]. The study concluded that ARO training significantly improves ophthalmoscopy skills compared to conventional training and suggested that subjective evaluation without systematic analysis should be avoided [27]. The study highlighted the benefits of ARO for beginners and medical students, noting advantages such as no need for pupil dilation, no light toxicity, and the availability of various retinal disease cases for training [27]. Despite the small sample size and lack of a crossover design, the results support the use of ARO in ophthalmology education [26].

Another study compared the traditional teaching approach of binocular indirect ophthalmoscopy (BIO) to the EyeSI AR BIO simulator in teaching novice residents [28]. This study was a prospective randomized control trial involving 28 postgraduate year one (PGY1) ophthalmology residents [28]. Fifteen residents were randomized to conventional teaching (group 1), which included a two-hour didactic lecture and practice on each other, followed by an evaluation on the EyeSI BIO simulator. Thirteen residents were randomized to AR simulator training (group 2) and directly completed training modules on the simulator before taking the evaluation. Three vitreoretinal fellows served as experts for comparison [28]. Outcome measures included total raw score, total time elapsed, and performance [28]. Following conventional BIO instruction, group 1 residents were outperformed by vitreoretinal fellows in all three outcome measures [28]. After completing the training modules on the simulator, group 1 residents showed significant improvement in elapsed time, total score, and performance compared to their baseline scores following conventional training only. Group 2 residents, who trained only on the simulator, demonstrated superior total scores and performance compared to group 1 residents after conventional training [28]. Once group 1 residents completed the AR BIO training, their performance was on par with group 2 residents. The study concluded that the EyeSI AR BIO simulator may be superior to conventional BIO teaching for novice ophthalmology residents [28].

On the other hand, another study by Petersen et al. examined whether pretraining of basic skills on a VR vitreoretinal simulator affects the performance curve when proceeding to procedure-specific modules [29]. A total of 68 medical students were included and equally randomized into two groups. This study was a prospective, randomized, controlled, two-center study involving medical students who were randomized into two groups. Group 1 was pre-trained in basic psychomotor skills (navigation training level 2 and bimanual training level 3) until they reached their performance curve plateau [29]. Both groups were then trained on the procedure-specific modules (posterior hyaloid level 3 and internal limiting membrane peeling level 3) until they reached their performance curve plateau [29]. Plateau was defined as three consecutive sessions with the same score within an acceptable variation. The primary outcome was the time used to reach the performance curve plateau in the procedure-specific modules [29]. The study concluded that there was no positive skill transfer from basic skills training to procedure-specific modules in terms of time, starting score, or amplitude of plateau [29]. Therefore, the study recommended that aspiring vitreoretinal surgeons proceed directly to simulation-based training of procedures instead of spending valuable training time on basic skills training [29].

Postoperative management

DTs can simulate a patient's recovery process post surgery by continuously integrating real-time data from wearable devices, EHRs, and other sources. This allows healthcare providers to monitor the healing process and anticipate potential complications before they manifest clinically. For example, in neurosurgery, DTs have been used to simulate the brain's response to different surgical interventions, helping predict postoperative complications like swelling or hemorrhage and allowing for early intervention [30].

A 2022 study by Lonsdale et al. discusses the use of human digital twins (HDTs) for perioperative monitoring in pediatric patients undergoing major scoliosis surgery. The study utilized wearable technology (a Fitbit Charge 3) to collect physiological data such as heart rate, step count, active minutes, and sleep patterns [31]. These data were used to create a baseline HDT for the patient, which allowed clinicians to monitor recovery and adjust postoperative care in real time [31]. The study demonstrates that HDTs can identify post-surgery variability and track when patients return to or exceed their preoperative baseline, providing valuable insights for guiding personalized rehabilitation strategies [31].

A 2021 study by Aubert et al. focuses on the development of patient-specific finite element models (FEM) to optimize trauma surgery and postoperative management, particularly for tibial plateau fractures [32]. This study utilized DTs to simulate different stabilization methods and bone healing scenarios, providing a detailed analysis of mechanical strength, stress distribution, and the risk of repeated fractures [32]. The DT allowed the surgical team to predict biomechanical outcomes and tailor postoperative care to the patient’s specific needs, such as determining the optimal time for weight-bearing and assessing the risks of complications [32]. The study highlights the potential of DTs to continuously monitor recovery and guide adjustments in postoperative care, ensuring better outcomes [32]. One of the key benefits of DTs in postoperative care is their ability to enable personalized adjustments to a patient's recovery plan. Continuous data integration from various sources, including physiological monitoring devices, allows clinicians to adapt care strategies in real time. For instance, a study on the use of DTs in cardiology highlighted how these virtual models could predict arrhythmias and guide postoperative care, such as adjusting medication dosages or altering rehabilitation exercises to optimize recovery [33].

Post-disease management

DTs are increasingly being employed to monitor long-term health outcomes and manage chronic diseases, such as cardiovascular disease (CVD) and diabetes. By integrating various data sources, including medical imaging, telemetry, wearable sensors, and genomic data, DTs can create dynamic models that track the progression of diseases like CVD. These models enable clinicians to monitor disease progression more effectively, predict complications, and provide timely interventions based on real-time data [34].

A study by Surian et al. highlights the use of DTs to monitor long-term health outcomes, particularly in chronic kidney disease (CKD) management for patients with type 2 diabetes mellitus (T2DM) [35]. The study developed a DT model using generalized metabolic fluxes (GMF) to simulate and predict CKD progression [35]. By integrating clinical and physiological biomarkers, the DT could identify CKD at baseline and predict disease progression over a three-year period [35]. This approach enables clinicians to monitor disease progression effectively and intervene early to prevent further complications [35].

The study by Wang et al. discusses the application of DT technology in healthcare, emphasizing its role in disease diagnosis, treatment, and monitoring [36]. Specifically, DTs are used to simulate diseased organs, predict the course of diseases, and monitor patient health in real time [36]. This technology is particularly beneficial for post-disease management as it allows for the continuous tracking of patient conditions, providing clinicians with insights into disease progression and potential complications [36]. The ability to monitor diseases dynamically enables more effective management of chronic conditions and improves the overall quality of patient care [36].

The study by Sun et al. delves into the application of DT technology in healthcare, emphasizing its potential in disease monitoring and management [7]. DTs act as virtual replicas of physical entities, allowing for real-time data collection and analysis, which is crucial for managing chronic diseases and monitoring post-disease recovery [7]. In particular, DTs can be used to simulate the progression of diseases and predict potential complications, enabling clinicians to monitor patients more effectively and make data-driven decisions about their care [7]. In postoperative and chronic disease management, DTs play a crucial role in facilitating personalized rehabilitation programs by simulating different recovery scenarios. For example, in diabetes management, DTs continuously update based on patient data, helping to simulate the impact of various treatment strategies on long-term health outcomes. This approach allows healthcare providers to tailor rehabilitation and recovery plans to the individual needs of each patient, thus improving overall outcomes [37].

DTs also play a crucial role in tracking recovery and facilitating personalized rehabilitation programs. The GMF-based DT model described by Surian et al. allowed for the stratification of patients into different risk groups based on their likelihood of CKD progression. This risk stratification was critical in guiding personalized rehabilitation strategies, as the DT could simulate the impact of various treatment scenarios and adjust rehabilitation programs accordingly [35]. This approach ensures that patients receive care tailored to their specific needs, improving overall recovery outcomes [35].

Wang et al. also highlight the potential of DTs in tracking recovery and facilitating personalized rehabilitation. By creating virtual models that mirror the patient's physiological state, DTs can simulate various recovery scenarios and help clinicians adjust rehabilitation programs based on real-time data [36]. This approach ensures that rehabilitation strategies are tailored to the specific needs of each patient, improving recovery outcomes and reducing the risk of complications during the rehabilitation process [36].

Chu et al. also highlight the role of DTs in tracking recovery and guiding rehabilitation efforts. By creating personalized models that mirror the patient's physical and physiological state, DTs can simulate various recovery scenarios [37]. This capability allows healthcare providers to tailor rehabilitation programs to each patient's needs, ensuring that interventions are adjusted in real time based on the patient's progress [6]. This personalized approach to rehabilitation can significantly improve recovery outcomes and reduce the likelihood of complications during the recovery process [7].

DTs also contribute significantly to refining long-term treatment strategies. In managing chronic diseases such as CVD, these virtual models can predict the effects of various treatments over time, enabling optimization of care plans. By utilizing real-time data and predictive analytics, DTs allow for continuous adjustment of treatment strategies, ensuring that they remain effective as the patient's condition evolves [34]. In optimizing long-term treatment strategies, the GMF DT model demonstrated its ability to predict CKD outcomes over extended periods, such as five and 10 years. This long-term predictive capability allows clinicians to refine treatment plans based on real-time data and adjust them as the patient's condition evolves. The study by Surian et al. underscores the potential of DTs in enhancing the precision and effectiveness of long-term disease management, ensuring that treatment remains aligned with the patient's changing health status [35].

In the context of optimizing long-term treatment plans, the study by Wang et al. emphasizes how DTs can be used to refine treatment strategies over time. By continuously integrating new data, DTs allow clinicians to adjust treatment plans as the patient's condition evolves, ensuring that care remains effective and aligned with the patient's changing needs [36]. This dynamic adjustment capability is crucial for managing chronic diseases and ensuring sustained positive health outcomes [36]. The study suggests that DTs are instrumental in refining long-term treatment plans. By continuously integrating real-time data, DTs allow for the dynamic adjustment of treatment plans as the patient's condition evolves. This ability to adapt and optimize care plans based on continuous feedback is crucial for managing chronic diseases and ensuring that treatment remains effective over the long term [7].

Digital twins' application case studies

Successful applications of DTs in post-disease management include managing diabetes and CVDs. In diabetes care, DTs have been used to create personalized models that help predict the efficacy of insulin regimens and lifestyle modifications, leading to better control of the disease [37]. Similarly, in cardiovascular care, DTs can simulate heart function to predict the outcomes of interventions like catheter ablation, guiding treatment decisions and improving patient outcomes [34].

The study by Surian et al. provides a case study in managing CKD among T2DM patients using DTs. The GMF-based model effectively identified high-risk patients for CKD progression and allowed for personalized care interventions [35]. By continuously monitoring metabolic changes and adjusting treatment plans, the DT model helped prevent the worsening of CKD in high-risk patients, demonstrating its utility in managing chronic conditions and improving long-term health outcomes [35].

The study by Wang et al. illustrates the broad applicability of DTs in post-disease management, from monitoring recovery to optimizing long-term treatment plans [36]. By providing a detailed and dynamic representation of a patient's health, DTs offer significant potential for improving patient outcomes through personalized, data-driven care [36].

Operations research

**Figure 4:**
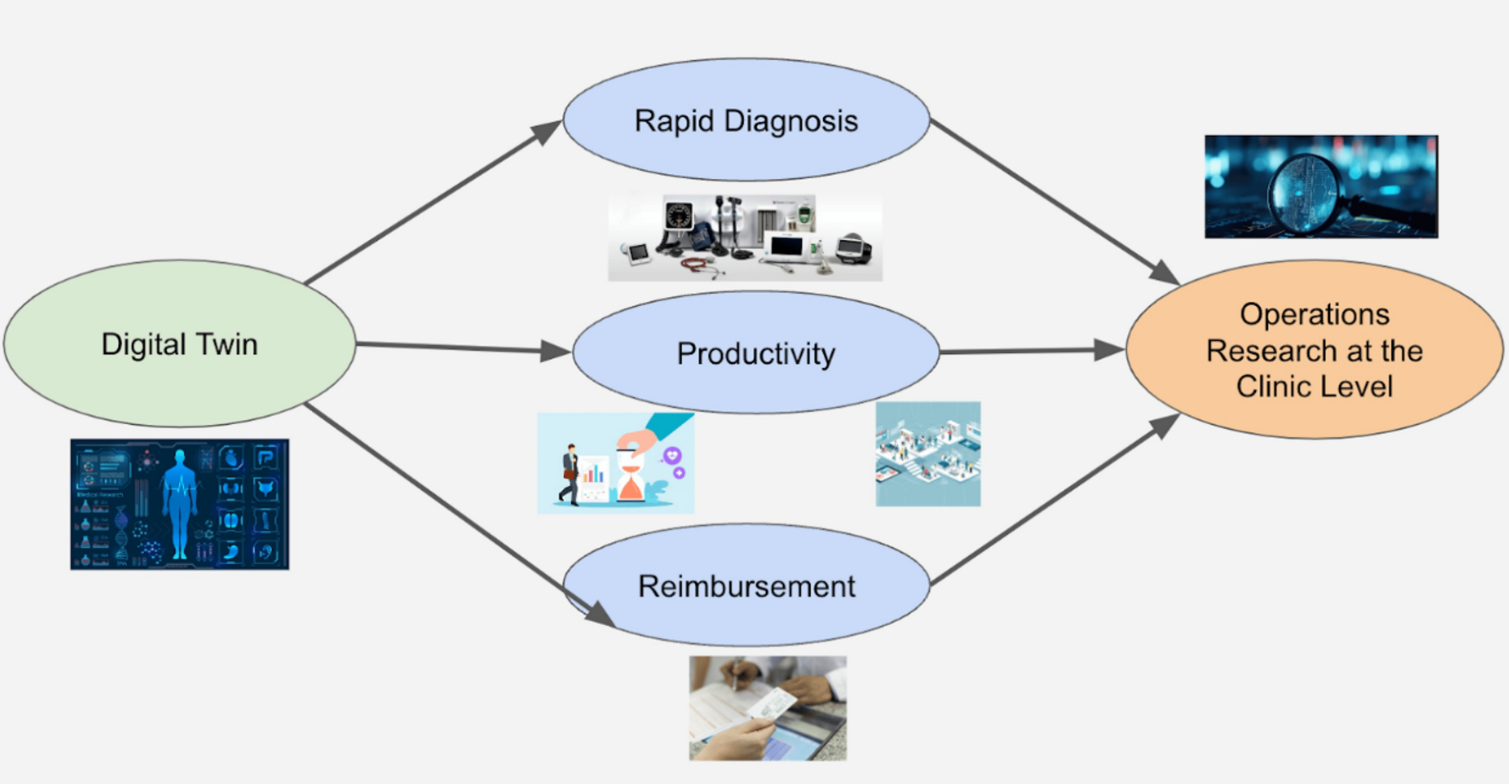
Digital twin integration in clinical operations research. Figure credit: Alfredo Paredes

One study by Pons evaluated how improving patient flow in eye clinics can enhance patient care and optimize the use of resources within the clinic. The study focused on analyzing patient flow in eye clinics by identifying various stages in a patient’s journey, from registration to treatment and follow-up [38]. It included assessing existing patient flow through audits, engaging both clinical and support staff to provide input, and regularly evaluating the flow to identify bottlenecks and areas for improvement [38]. Furthermore, the study also examined practical suggestions for better use of space, staff, and systems to improve patient flow, shown visually in Figure 4 [38].

The findings highlighted that optimizing patient flow could significantly improve the patient experience by reducing waiting times, eliminating unnecessary steps, and making better use of clinic resources [38]. Patients value timely and efficient care, appropriate referrals, correct diagnoses, and adequate information and follow-up [38]. The study found that addressing bottlenecks, such as long queues at visual acuity testing stations, and making systematic changes, like arranging patient stations logically and utilizing color-coded lines for direction, could enhance patient flow [38]. Additionally, improving internal communication systems and making better use of mid-level ophthalmic personnel and nursing staff could further streamline clinic operations [38]. The study concluded that focusing on patient flow in eye clinics not only improves the patient experience but also benefits the clinic by making more efficient use of resources and reducing costs [38]. Practical changes, such as optimizing the layout of clinic stations, improving communication, and involving all staff in the process, can lead to significant improvements in both patient care and clinic operations [38]. The ongoing evaluation and adjustment of patient flow are crucial for maintaining efficient and effective eye care services [38].

Another modality of virtual employment to improve operations research was examined in a study by Atta et al., which assessed the efficacy of EHR messaging in re-engaging patients with ophthalmology care after they missed an appointment [39]. Over two phases of recruitment, 362 patients were selected and randomized into two groups: one group received a standard mailed letter only (control), and the other group received the mailed letter plus an electronic message through the EHR within one business day of the missed appointment (intervention) [39]. The primary outcome was re-engagement with eye care, defined as attending a rescheduled appointment within 30 days of the no-show visit [39]. The average age of the participants was 59.9 years, with just under half being male (42.5%) and most belonging to White (56.9%) or Black (36.2%) race [39]. Patients were most commonly recruited from the retina (39.2%) and glaucoma (29.3%) services [39]. The intervention group had a significantly higher rate of attending a follow-up appointment within 30 days (22.2%) compared to the control group (11.6%) (OR, 2.186; 95% CI, 1.225-3.898; P = 0.007) [39]. When considering only those who read the message, 28.4% of the intervention group attended a follow-up compared to 11.6% of the control group (P = 0.001) [39]. The study concluded that EHR-based reminder messages sent within one business day of a missed appointment significantly improved re-engagement in ophthalmology care, doubling the likelihood of patients attending a rescheduled appointment within 30 days [39]. The findings suggest that such interventions can be a valuable tool in reducing no-show rates and improving patient care outcomes in ophthalmology settings [39].

DTs allow for the simulation of real-world systems under various conditions, enabling operations researchers to optimize processes and workflows. By creating a virtual replica of a physical system, researchers can test different scenarios and strategies without disrupting actual operations. This is particularly beneficial in manufacturing and supply chain management, where optimizing logistics and production processes can lead to significant cost savings and efficiency improvements [40,41]. In operations research, DTs are crucial for predictive maintenance. These virtual models continuously monitor the performance of machinery and systems, predicting when and where failures are likely to occur. By enabling pre-emptive maintenance, DTs reduce downtime and extend the lifespan of equipment. This application is widely used in industries like aerospace and automotive manufacturing, where equipment reliability is critical [42]. DTs enhance supply chain management by providing a comprehensive view of the entire network, from production to delivery. Operations researchers can use these models to simulate disruptions, forecast demand, and optimize inventory levels. This capability improves decision-making and ensures that the supply chain operates smoothly, even under unexpected conditions [41,43].

Discussion

Galar et al. discuss how DTs can optimize resource utilization and reduce unnecessary medical procedures, leading to considerable cost savings in healthcare. They emphasize that DTs can enhance process efficiency, improve patient outcomes, and decrease the incidence of medical errors [44]. By providing accurate simulations and predictions of patient conditions, DTs enable more informed decision-making, which translates into lower healthcare costs and better resource management [44]. This study also highlights the potential for DTs to reduce operational costs in areas such as surgery and chronic disease management, making them a valuable tool for healthcare providers [44].

Venkatesh et al. discuss the economic implications of implementing DTs in healthcare, particularly focusing on precision medicine. The study outlines how DTs can lead to significant cost savings by reducing unnecessary medical procedures, optimizing resource utilization, and improving overall treatment outcomes [9]. The potential financial benefits include reduced healthcare costs through more accurate diagnostics and targeted therapies, as well as increased efficiency in patient management [9]. Moreover, the study suggests that the adoption of DTs could be incentivized through innovative payment models that reward improved patient outcomes, making the investment in such technology more appealing to healthcare providers [9].

Sun et al. explore the financial impact of DTs, emphasizing their role in personalized medicine. The study suggests that DTs can significantly reduce healthcare costs by improving the accuracy of diagnoses and the efficiency of treatment plans [7]. This is particularly relevant in chronic disease management, where continuous monitoring and predictive analytics can prevent costly complications [7]. The paper also highlights the potential for DTs to streamline clinical trials and regulatory processes, further reducing the time and cost associated with bringing new treatments to market [7].

Kshetri provides a comprehensive overview of the economic benefits of DTs across various industries, including healthcare. The paper discusses how DTs can improve operational efficiency, reduce costs, and manage risks more effectively [45]. In the healthcare sector, DTs are particularly valuable in reducing the time and cost associated with medical product development and regulatory approvals [45]. The study also highlights the broader economic impact of DTs, noting that the global market for this technology is expected to grow significantly, driven by advancements in AI, IoT, and big data analytics [45].

Iliuță et al. examine the financial implications of DTs in ophthalmology. The study suggests that integrating DTs in ophthalmic practice can lead to significant cost reductions by improving disease management and reducing the need for costly medical interventions through early detection and optimized treatment strategies [13]. However, the study also notes the substantial initial costs associated with acquiring the necessary infrastructure, data management systems, and training personnel [13]. Despite these costs, the long-term financial benefits, such as decreased treatment costs and improved patient outcomes, are considered to outweigh the initial investment [13].

Proposed Framework for Digital Twin Application

To begin a proposed framework for DT, patient-specific data, including genetic, demographic, lifestyle, and detailed medical history, are gathered to create an initial model of the patient’s health profile. Ophthalmology-specific data, such as retinal images, intraocular pressure, and OCT images, are prioritized as essential inputs for understanding both baseline health and potential disease progression. This foundation is enhanced by real-time data collection through wearable devices or periodic eye scans, allowing for the continuous intake of physiological information and establishing a rich historical data layer [46]. This dataset offers a high-resolution snapshot of the patient’s ocular condition, capturing both stable traits, like genetic predispositions, and dynamic metrics, such as blood glucose levels in diabetic patients, that influence disease risks and treatment outcomes [46].

Within this framework, convolutional neural networks (CNNs) play a central role in disease classification by analyzing retinal images in real time. As demonstrated in studies utilizing CNN models such as VGG-16 and ResNet, CNNs are adept at accurately categorizing OCT images into distinct classes, including “normal,” “CNV” (choroidal neovascularization), “DME” (diabetic macular edema), and “drusen,” which are indicative of early macular degeneration [47]. In this context, CNNs provide the DT with high-accuracy classifications that serve as primary diagnostic markers, supporting immediate and ongoing assessments of the patient's condition. For instance, in a diabetic retinopathy case, the CNN would analyze retinal images to identify key early indicators, such as microaneurysms, hemorrhages, and exudates, that could signify disease onset or progression. This capability facilitates precise identification and allows the DT to simulate disease progression under various scenarios [47]. The DT’s predictive modeling capabilities are then employed to forecast disease development over time. By simulating hypothetical scenarios based on the patient’s specific genetic, clinical, and lifestyle data, the DT can project how conditions like diabetic retinopathy or glaucoma may progress, offering predictive insights that allow for proactive healthcare management [48]. The DT’s integration with machine learning algorithms enhances its ability to forecast disease trajectory with high specificity by incorporating multiple data dimensions, such as a patient’s historical health patterns, OCT image analysis, and lifestyle influences like smoking or diet. This predictive aspect is essential, especially for chronic diseases that benefit from early intervention, as it provides clinicians with reliable foresight into potential disease paths [48].

Moreover, the DT framework offers robust decision support for clinicians. Through the continuous collection and analysis of patient data, it can generate highly personalized treatment plans that evolve with the patient’s needs. For example, a patient with diabetic retinopathy who presents a high genetic risk for disease progression could benefit from a tailored treatment regimen that includes optimized drug dosages, lifestyle modifications, and regular retinal monitoring [49]. The DT, through its CNN integration, allows the clinician to test potential interventions virtually before they are applied, thus minimizing adverse side effects and ensuring the treatment plan is ideally suited to the patient's unique health profile [49]. As the DT assimilates new data from follow-up exams or wearable devices, it recalibrates its simulations, offering updated treatment recommendations that reflect the patient’s evolving health status. This adaptive feedback loop reduces the likelihood of treatment resistance or disease relapse, making it particularly valuable in managing progressive conditions like diabetic retinopathy [49].

A crucial component of this framework is its real-time adaptability, where continuous updates from patient exams or wearable monitoring devices enable the DT to adjust predictions and recommendations dynamically. For instance, if a patient’s retinal scans reveal signs of macular edema progression, the DT can immediately simulate alternative treatments and drug adjustments, providing clinicians with an evidence-based guide to modify the current treatment plan effectively. This dynamic approach enhances the precision of disease management by aligning the treatment protocol with the patient’s most recent health indicators, ensuring that care decisions are both timely and highly individualized. The framework also prioritizes patient engagement, using DT visualizations to show how lifestyle modifications or consistent treatment adherence may alter disease trajectory. For example, a patient at high risk for age-related macular degeneration could view simulations that depict the long-term impact of factors like smoking cessation or dietary adjustments on disease outcomes. By empowering patients with a visual understanding of their condition, the DT framework fosters a collaborative healthcare experience where patients are informed participants in their treatment journey.

Implementing DT technology in healthcare presents significant challenges, particularly in terms of cost, training, and resistance to adoption. From a financial perspective, the initial investment required for DT infrastructure is substantial, including the acquisition of IoT devices, advanced computational systems, and AI algorithms for predictive modeling. For instance, Iliuță et al. emphasize the high costs associated with integrating DTs into ophthalmology, particularly for managing chronic diseases like glaucoma, where sophisticated data collection and processing systems are required to simulate real-time patient scenarios. Despite the initial expense, long-term financial benefits have been demonstrated. DTs can reduce overall healthcare costs by minimizing unnecessary procedures, streamlining diagnostics, and personalizing treatments. For example, Venkatesh et al. estimate that DT-driven decision-making in chronic disease management could save millions annually by targeting therapies with higher efficacy and fewer complications [9]. Governments and healthcare organizations could explore subsidizing infrastructure costs or implementing public-private partnerships to facilitate the initial deployment of DT systems.

In terms of training, the adoption of DTs necessitates equipping healthcare professionals with the skills to operate and interpret complex digital models. Training programs incorporating immersive simulation environments have proven effective in surgical applications, where clinicians can practice procedures in virtual settings, reducing errors and improving patient safety. For example, orthopedic surgeons using DT-based simulations to plan trauma surgeries reported significant reductions in intraoperative complications. To overcome training gaps, healthcare institutions should implement structured training modules, including workshops and certification programs, to familiarize staff with DT systems. Collaboration with academic institutions could further standardize training curriculums for medical professionals.

Resistance to adoption often stems from a lack of familiarity with new technologies and skepticism about their efficacy. Successful applications of DTs in fields outside ophthalmology, such as prostate cancer treatment and CVD management, demonstrate their potential to improve outcomes. In prostate cancer, DTs enabled personalized treatment plans that optimized tumor control while minimizing side effects, reducing apprehension among healthcare providers about adopting these tools. To address resistance, healthcare organizations must demonstrate the tangible benefits of DTs through pilot programs and case studies, showcasing real-world success in improving patient outcomes and streamlining workflows. Additionally, engaging stakeholders, including clinicians, patients, and policymakers, in discussions about the potential of DTs can help alleviate concerns and build trust in the technology.

Limitations

The limitations of the study are multifaceted and stem from both methodological and contextual factors. The reliance solely on PubMed, IEEE (Institute of Electrical and Electronics Engineers), and Web of Science for sourcing literature, while ensuring reputable sources, may have excluded relevant studies from other specialized or interdisciplinary databases, potentially limiting the scope of the review. Additionally, the inclusion criteria required studies to integrate genetic, demographic, and physiological data and demonstrate relevance to ophthalmology-specific applications, such as predictive modeling, disease management, and personalized medicine. While this approach provided valuable insights into diverse applications, the variability in methodologies and applications across the selected studies introduced inconsistencies that complicate generalizability. The lack of uniform metrics for evaluating DT technology further hinders the ability to systematically compare findings and draw cohesive conclusions. The review also highlighted challenges in data integration and patient privacy, which are critical barriers to the effective implementation of DTs, but it did not explore these issues in depth.

Another limitation is the emerging nature of the field itself, with many studies focusing on experimental frameworks or small-scale applications, making it difficult to apply findings broadly to clinical practice. The substantial initial investment required for infrastructure, training, and data management systems poses a significant barrier to adoption in clinical settings. Lastly, the geographic and socioeconomic representation in the reviewed studies may predominantly reflect high-resource settings, potentially overlooking challenges and innovations in low-resource or diverse contexts. Moreover, there are limited large-scale clinical trials validating the efficacy of DTs, the high variability in data integration and standardization across studies, potential biases from small sample sizes and publication preferences, and the absence of cost-effectiveness analyses over long-term implementations pose weaknesses and hinder the current reality of DT in ophthalmology. Additionally, there is a limited exploration of ethical considerations, interoperability challenges, and the scalability of DT applications in diverse healthcare settings.

Future Direction

Future research on DTs in healthcare should focus on overcoming current limitations and expanding their applications, particularly in ophthalmology. A critical area for development is the integration of multimodal data sources, such as genetic profiles, environmental factors, patient history, and real-time monitoring data. Studies should assess the efficacy of algorithms in harmonizing these data types to create robust and dynamic patient models. Additionally, advancements in predictive modeling are essential; research should refine machine learning algorithms to improve the accuracy of disease progression simulations and treatment outcome predictions across diverse patient demographics. Large-scale clinical trials are necessary to validate DTs’ efficacy in improving outcomes, ensuring the technology is generalizable across various populations and conditions. Evaluating the long-term cost-effectiveness of DTs is also vital, with a focus on analyzing cost savings through reduced hospitalizations, optimized treatment plans, and improved resource allocation. Financial models should explore reimbursement structures and incentives for healthcare providers adopting DT technology.

Ethical considerations must be addressed, particularly regarding patient consent, data ownership, and privacy. Research should develop standardized protocols to manage sensitive patient data securely. The impact of clinician training programs for DT implementation should also be studied to evaluate how these tools influence decision-making and workflow efficiency. Understanding barriers to adoption, such as resistance to new technologies and the associated learning curve, is critical for targeted solutions. Key metrics for assessing the overall viability of DTs include accuracy in predicting disease progression and treatment outcomes, measurable improvements in patient recovery rates and quality of life, reductions in resource use and hospital stays, financial impact analyses over short- and long-term periods, and satisfaction ratings from both clinicians and patients. Addressing these aspects will ensure the refinement of DTs and support their adoption as a transformative tool in healthcare delivery.

## Conclusions

This study highlights the transformative potential of DT technology in ophthalmology, emphasizing its capacity to deliver personalized, data-driven care. By integrating genetic, environmental, and real-time patient data, DTs provide a comprehensive view of a patient’s health, allowing for more accurate risk assessments, improved disease management, and optimized surgical planning. The ability of DTs to simulate various treatment scenarios and predict outcomes enables clinicians to make more informed decisions, ultimately leading to better patient outcomes. However, the successful implementation of DTs in healthcare, particularly in ophthalmology, requires overcoming several challenges, including data integration, privacy concerns, and substantial initial investment in infrastructure and training. Despite these hurdles, the long-term benefits, such as cost savings through reduced unnecessary procedures, improved resource utilization, and enhanced patient care, make DTs a valuable tool in the future of personalized medicine. As technology advances and the adoption of DTs becomes more widespread, their impact on healthcare is expected to grow, offering new possibilities for precision medicine and patient-centered care.
